# Highly Defined, Colloid-Like Ionic Clusters in Solution

**DOI:** 10.1002/open.201200025

**Published:** 2012-09-05

**Authors:** Dennis Kurzbach, Daniel R Kattnig, Nane Pfaffenberger, Wolfgang Schärtl, Dariush Hinderberger

**Affiliations:** a Max Planck Institute for Polymer ResearchAckermannweg 10, 55128 Mainz (Germany) E-mail: dariush.hinderberger@mpip-mainz.mpg.de; b Institute for Physical Chemistry, Johannes Gutenberg-University MainzJakob-Welder-Weg 11, 55099 Mainz (Germany)

**Keywords:** dynamic light scattering, molecular dynamics, Monte Carlo simulations, nanoscale electrostatics, self-assembly

Many societal challenges at the beginning of the 21st century lead to an apparent and growing need for functional materials and novel ways of materials synthesis and assembly. Rising to the challenge, the utilization of small, self-assembling building blocks for the bottom-up construction of new types of polymers and nanostructures has enjoyed increasing popularity among materials researchers in the recent past. Supramolecular materials like foldamers, surface films, nanoparticles, etc. are created by exploiting noncovalent forces[1] leading to an ordered arrangement of nanoscale building blocks.[2]

In the search for new polymers based on noncovalent molecular forces, we are motivated by the idea of supramolecular or even polymer-like structures by self-assembly of small ionic monomers, merely formed from electrostatic and solvent interactions. We, in particular, focus on applying intrinsically nondirected electrostatic interactions to create nonetheless well- organized supramolecular structures.[3] To achieve this aim, we here make use of a recently highlighted, multicationic molecular box (**1**^4+^; Figure 1 a), developed by Sessler and co-workers, which has already been applied in self-assembly applications of metal–organic rotaxane frameworks.[4] The molecular box **1**^4+^ seems to facilitate structure generation through self- assembly, indicated by, for example, the surprising ability to build supramolecular necklaces through incorporation of electron-rich aromatic guest molecules.[5] However, not focused on short-ranged interactions but on new applications of long-ranged electrostatic forces at the nanoscale,[6] we combine **1**^4+^ with small di-anionic salts, (KSO_3_)_2_CH_2_ (**2**^2−^) and (KSO_3_)_2_NO (**3**^2−^), to generate a defined assembly of cations and anions in solution (Figure 1 a). However, self-assembly due to electrostatic attraction is complex to describe qualitatively as well as quantitatively, because not only Coulomb forces but also entropy changes due to counterion release, solvation of ions, depletion forces, etc., contribute to the total free energy of a system. A central question in any application of solution-based ionic self-assembly therefore is: does any kind of ordered arrangement of the ionic building blocks take place?[7] Intuitively one would assume the distribution of ions in solution to be of random, rather homogeneous nature.

**Figure 1:**
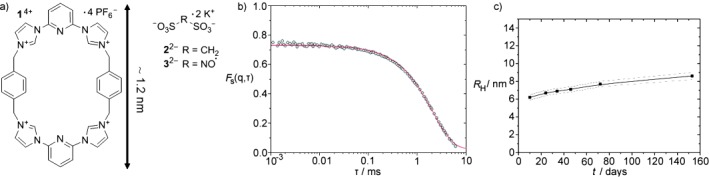
a) Molecular structure of **1**^4+^ (left) and **2**^2−^/**3**^2−^ (top right). b) A typical monoexponential fit of an autocorrelation function gained from DLS on **2**^2−^/**1**^4+^ *ionoids*. c) Hydrodynamic radii of the **2**^2−^/**1**^4+^ *ionoids* plotted against time, as derived from DLS measurements. *t*=0 days refers to the date of initial preparation. The upper and lower lines indicate error estimates derived from the fits of the autocorrelation functions.

In the following, we show dynamic light scattering (DLS) data, which clearly illustrate that low-concentrated mixtures of anionic **2**^2−^ and cationic **1**^4+^ self-assemble in solution to highly defined, monodisperse ionic clusters. In this special case, the distribution of ions in solution is therefore not at all random but well-ordered due to long-range electrostatic correlations. Performing molecular dynamics (MD) and Monte Carlo (MC) simulations, we further elucidate the internal constitution of the ionic clusters and show that a long-ranged order may be induced by electrostatic correlations of the cationic macrocycles. The simulations indicate that the monodisperse objects are constituted of relatively loosely bound ions that, however, occupy certain fixed coordinates in the clusters for at least orders of nanoseconds. The reliability of the computational data is additionally supported by double electron–electron resonance (DEER)[8] spectroscopy data. Taking long-ranged (DLS, MC) and molecular (MD, DEER) insights together, we demonstrate, to the best of our knowledge for the first time, that size-controlled self-assembly through electrostatic interaction between small ions in solution is possible. The so formed objects can be regarded as loosely bound ion-based colloids, thus we propose the name *ionoids*. These *ionoids* are peculiar as they result from ion–ion correlations of moderately charged constituents at low concentrations, while long-range electrostatic correlations have so far only been discussed in the context of macroions.[9]

DLS experiments were performed on a system containing a mixture of **2**^2−^/**1**^4+^ at an approximate ratio of 3 mm:1 mm in DMSO/88 % aqueous glycerol (1:1). Note that the exact concentration of **2**^2−^ within the light-scattering sample is difficult to determine, as the solubility of the pure component **2**^2−^, in contrast to **3**^2−^, is only 3 mm in our solvent mixture. The measurements revealed monodisperse small objects in the solution, after an initial period of several days where no correlation could be detected. The fit of one correlation function measured at a scattering angle of 90 ° is depicted in Figure 1 b. Note the monoexponential nature of this fit, which indicates monodispersity of the aggregates in the solution. The intercept of the correlation function is unusually low, due to the small signal-to-noise ratio of <3, that is, the scattered intensity from the pure solvent mixture is nearly 40 % of the overall scattered intensity. As, in the absence of **2**^2−^_,_ we found no correlation at all but only the DLS signature of the pure solvent mixture, it is at hand to deduce that these small objects stem from self- assembly of **2**^2−^–**1**^4+^ clusters. The hydrodynamic radius of the self-assembled **2**^2−^–**1**^4+^ cluster is quite small, that is, only 6.2 nm±0.2 nm can be observed 10 days after sample preparation, thus, growing very slow (+2 nm after 140 days; see Figure 1 c) and fostering an analogy with charge-stabilized colloids. It is important to note that all correlation functions measured over time can be fitted by a single exponential decay, and the resulting diffusion coefficient is independent of the scattering angle, which underlines the monodispersity in aggregate size. Thus, one can conclude that molecules **2**^2−^ are necessary to overcome like-charge repulsion and to bring together 3–4 molecules **1^4+^** (estimated from the size of approx. 6–7 nm) in an extraordinarily uniform manner.

To support the reliability of the DLS data and to elucidate the internal constitution of the ionic clusters, we conducted all-atom MD simulations corresponding to a **3**^2−^/**1**^4+^ system at a molar ratio of 6:2. In order to check the reliability of the MD simulation, we supplement it with DEER data and therefore replaced **2**^2−^ by paramagnetic **3**^2−^. (This change may have an influence on special interactions in the *ionoids*, yet long-range electrostatic interactions remain unchanged. Note that the concentration in the simulations was three times higher than in the DEER measurements, to reduce the computation time). Starting from a random distribution of all molecules, we let the system equilibrate for 0.2 ns, and subsequently we extracted the trajectories for 8 ns with the aim of calculating the radial distribution function, *g*(*r*), of **3**^2−^. Figure 2 a shows a snapshot of the simulation after 2 ns, illustrating the molecular arrangement of the ions in such an *ionoid*-type cluster. Figure 2 b depicts the radial distance distribution of **3**^2−^ summed up over 8 ns of simulation and over different time intervals of 1 ns. Prominent features in the overall *g*(*r*) at 1.9 nm, 2.4 nm, 3.5 nm are observable. Thus, the distribution of **3**^2−^ in the simulated system is far from being random. Also, the distributions summed up in each case over 1 ns of simulation indicate favored population of distinct distances in the **3**^2−^/**1**^4+^ system. At first sight, it is astonishing that the maxima in *g*(*r*) occur with a spacing of approximately 0.5 nm, indicating a periodic arrangement of the spin probes (Figure 2 b).

**Figure 2:**
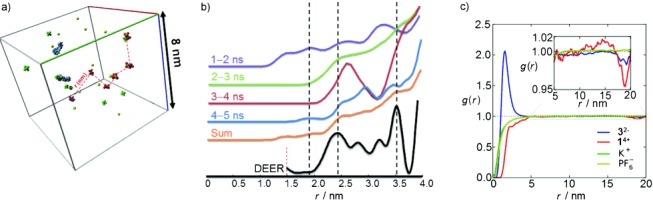
a) Snapshot after 2 ns of MD of the system **3**^2−^/**1**^4+^ at 6:2 molar ratio. b) Pair correlation function of **3**^2−^ molecules gained from MD simulation of the system **3**^2−^/**1**^4+^ (6:2) summed up over different time intervals. The overall distance distribution is shown in orange (—). Note that over time, certain maxima in the distributions appear frequently, as indicated by the dashed lines. The black distribution depicts the distances gained from DEER on the same system (—). c) The Monte Carlo-derived radial distribution function for **3**^2−^ calculated for two **1**^4+^ and six **3**^2−^ molecules (—). The box size was 26^3^ nm^3^. As can be seen, there are no distinct maxima in the distance distribution. Hence, the aggregation of **3**^2−^ and **1**^4+^ is not based on Coulomb interaction and excluded volume effects alone. Solvent entropy may possibly contribute significantly to the generation of the *ionoids*, as the MD-derived distributions lead to reasonable agreements with the experimental data. The **1**^4+^–**1**^4+^ distribution (—) reveals a long-range correlation at approximately 15 nm. For more details, see the Supporting Information.

Although the inner constitution of the **3**^2−^–**1**^4**+**^ aggregates could be assessed with MD, the global size of the so formed aggregates cannot be deduced from the simulation and compared with *R*_H_ because of a box size of only 8^3^ nm^3^. Consequently, we performed Monte Carlo (MC) simulations with a box size of 26^3^ nm^3^ to elucidate the individual cluster size. These simulations included electrostatic potentials as well as excluded volume effects. However, the MC simulation showed that, due to mere electrostatic interactions and excluded volume, no distinct short-range association takes place (Figure 2 c). Yet, the MC simulations do reveal a surprising long-range correlation of **1**^4+^ at about 15 nm, suggesting a defined size of the observed ionic clusters, electrostatic and excluded volume effects to be essential for the formation of the observed ionic clusters (Figure 2 c and the Supporting Information), because the experimentally determined radius (6.2 nm–8.2 nm) and the size suggested from MC simulations (*R*≍7.5 nm) are in good agreement. Yet the distinct **3**^2−^–**3**^2−^ distances, as gained from MD, seem to be a consequence of additional entropic or solvent effects next to mere electrostatic effects.

To further support the reliability of the computational data, we performed electron paramagnetic resonance (EPR)-based nanoscale distance measurement (DEER) experiments on a freeze-quenched **3**^2−^/**1**^4+^ system to extract the experimental distance distribution of **3**^2−^. The resulting distance distribution is depicted in Figure 2 b (black curve). It shows the population of all **3**^2−^–**3**^2−^ distances of 6 mm molecules **3**^2−^ around 2 mm molecules **1**^4+^ and can be compared directly with the *g*(*r*) gained from MD. Taking into account that the simulation features significantly higher concentrations than the experimentally measured system (the **1**^4+^ concentration was three times higher than in the experiment to increase the speed of calculation) and the restricted number of simulated **1**^4+^ and **3**^2−^ molecules, the simulation-derived and the experimental distribution overall are in reasonable agreement. However, there are no distinct dipolar modulations observable in the DEER time traces (see the Supporting Information). Hence, the extracted distances (by Tikhonov regularization) have to be treated with caution. Yet, as they are clearly indicative of nonhomogeneous/nonrandom distributions of **3**^2−^ and in addition are even in reasonably good agreement with the simulated distributions, one can assume that the distances extracted from the MD simulation resemble the situation inside the *ionoids* to a certain degree. Thus, judging from the simulation, the situation as shown in Figure 2 a can be taken as a representative snapshot of the distribution of ions in the solution. As it does not change significantly over time, it seems like the molecules **1**^4+^ are forced into a short-distance arrangement in order to minimize the potential electrostatic energy of the whole system.

Divalent molecules **3**^2−^, thereby, could be acting as ‘ionic glue’ between macrocycles **1**^4+^. In doing so, the positions of local maxima in the distance distribution as derived from the MD simulation do not change significantly over time. This means that the spatial conformational ensemble covered seems to be relatively small. Once **3**^2−^ is ‘trapped’ in an ensemble, it remains at a certain position for at least several nanoseconds. Continuous wave (CW)-EPR data at room temperature heavily support this hypothesis: the **3**^2−^ probes still rotate relatively freely, although their rotational mobility is slightly restricted when small amounts of **1**^4+^ are added to a **3**^2−^ solution (see the Supporting Information for the CW-EPR spectra and corresponding spectral simulations). At the same time, they still favor certain coordinates in the *ionoids* because of electrostatic attraction to **1**^4+^ and repulsion among themselves. It is likely that the combination of larger organic and small, yet doubly charged, inorganic building blocks is necessary for the emergence of ordered ionic assemblies, which persist in solution over time. Also, solvent effects could play a role, as DMSO is known to feature anomalous properties because of self-association.[10] Taking all the experimental and computational data into account, one can conclude that the self-assembled objects feature a defined radius, although, on a molecular scale, their interior seems not to be strongly fixed: ‘gluing’ **3**^2−^ molecules can still rotate relatively undisturbed. Yet, on the supramolecular scale, certain defined coordinates inside the aggregates are preferentially populated for at least some nanoseconds. Hence, the position of the ‘glue′ is not arbitrary. From a physico-chemical point of view it is highly intriguing that an intrinsically nondirected force like electrostatic attraction/repulsion leads to the formation of highly defined nanoscopic objects. One can even control the initial size of the self-assembled *ionoids* precisely and keep them stable in solution for months. Thus, by choosing the right ions and counterions, their distribution in solution is not more or less homogeneous as one would expect intuitively for electrolyte solutions.[10–11] Contrarily, they assemble into discrete arrangements as has been observed for macroions such as in DNA-condensation years ago.[12]

As the observed *ionoids* are based on noncovalent interaction among constituents, they probably are sensitive towards even minor changes in the solvent properties, ionic strength of the solution, and concentration of the constituents. These factors are likely to influence their composition and size. Furthermore, we expect that the observed growth of the *ionoids* will be fostered by a decrease in solvent viscosity, and that their occurrence is limited to certain solvents.

Altogether, we have presented experimental and computational evidence for the formation of electrostatically self-assembled supramolecular structures in solution. The nano-assemblies have a well-defined size, and molecules in their interior populate, at least transiently, certain fixed positions in the cluster, while keeping a certain rotational freedom. The so formed *ionoids* can possibly open new routes towards the design and self-assembly of novel materials by exploiting electrostatic interactions between small molecules which have not attracted much attention in this field of research so far. In addition, these nano-objects are intriguing from a theoretical point of view, as long-range electrostatic correlations in solution have been postulated but, to the best of our knowledge, have not yet been observed directly for low-molecular electrolytes (unlike macroions).[13] From the slow but steady size increase over the course of two months (Figure 1 c) one could even speculate that *ionoids* could be some sort of solvated precursor for precipitation or even crystallization nuclei. More experimental evidence would be needed to further substantiate such a picture, which currently is a mere speculation. Considering the small size of the *ionoids*, it is likely that X-ray and neutron scattering experiments will yield substantial insight into the specific properties of these solution structures. Such measurements are currently on our agenda. The responsiveness of *ionoids* towards stimuli such as concentration, solvent-mixture or charge ratio could also become interesting in the course of the search for new responsive materials.

## Supplementary material

Detailed facts of importance to specialist readers are published as ”Supporting Information”. Such documents are peer-reviewed, but not copy-edited or typeset. They are made available as submitted by the authors.
