# Supplementary material for: Highly Defined, Colloid-Like Ionic Clusters in Solution
Source: ChemistryOpen. 2012 Sep 5;1(5):211–4. doi: 10.1002/open.201200025 (PMC3922591; doi:10.1002/open.201200025)
Supplement: Supplementary file 1 [file open0001-0211-SD1.pdf]

ChemistryOPEN

Supporting Information

© 2012 The Authors. Published by Wiley-VCH Verlag GmbH & Co. KGaA, Weinheim

Highly Defined, Colloid-Like Ionic Clusters in Solution

Dennis Kurzbach,[a] Daniel R. Kattnig,[a] Nane Pfaffenberger,[b] Wolfgang Schärtl,[b] and Dariush Hinderberger*[a]

open_201200025_sm_miscellaneous_information.pdf

CW EPR Measurements

A Miniscope MS200 (Magnettech, Berlin, Germany) benchtop spectrometer was used for X-band CW EPR measurements at a microwave frequency of ~9.4 GHz. Measurements were performed at room temperature (293 K) using a modulation amplitude of 0.05 mT. The microwave frequency was recorded with a frequency counter (Racal-Dana, model 2101).The temperature was set with a HO2-temperature control unit.

All spectral simulations were performed with home-written programs in MATLAB (The MathWorks, Inc.) employing the EasySpin toolbox for EPR spectroscopy.[1] Simulations of CW EPR spectra in fluid solution were performed by using a model, which is based on the slow-motion theory developed by Schneider and Freed as implemented in EasySpin.[2] These simulations can account for the effect of intermediate or slow rotational diffusion of the radical on the EPR timescale. All reported values for hyperfine-coupling parameters and rotational correlation times were obtained from simulating the experimental CW EPR spectra.

DEER Measurements

DEER is applied to glassy solids obtained by freeze-quenching the ionoid solutions in supercooled iso-pentane. In this way a snapshot representative for the solution at the glass-transition point is detected. The sample volume was always large enough to fill the complete resonator. The four pulse DEER sequence $\pi/2(\nu_{obs})$ - τ_1 - $\pi(\nu_{obs})$ - $(\tau_1 + t)$ - (ν_{pump}) - $(\tau_2 - t)$ - $\pi(\nu_{obs})$ - τ_2 - echo was used to obtain dipolar time evolution data at X-band frequencies (9.2 to 9.4 GHz) with a Bruker Elexsys 580 spectrometer equipped with a Bruker Flexline split-ring resonator ER4118X_MS3. The dipolar evolution time t was varied, whereas τ_2 = 3 µs and τ_1 were kept constant. Proton modulation was averaged by the addition of eight time traces of variable τ_1, starting with $\tau_{1,0}$ = 200 ns and incrementing by $\Delta\tau_1$ = 8 ns. The resonator was overcoupled to Q ≈ 100. The pump frequency, ν_{pump}, was set to the maximum of the EPR spectrum (Figure S1b)). The observer frequency, ν_{obs}, was set to ν_{pump}+ 57.4 MHz, coinciding with the low field local maximum of the nitroxide spectrum (Figure S1b)). The observer pulse lengths were 32 ns for both π/2 and π pulses, and the pump pulse length was 12 ns. The temperature was set to 50 K by cooling with a closed cycle cryostat (ARS AF204, customized for pulse EPR, ARS, Macungie, PA). The total measurement time for each sample was around 12 h. The raw time domain DEER data were processed with

the program package DeerAnalysis2010.[3] Background-contributions were removed dividing the time traces by an experimental background function (dimensionality = 3), derived from DEER on 6 mM $\mathbf{3}^{2-}$ solutions. The resulting time traces were normalized at t = 0.

Extraction of Distance Distributions from DEER Time-Traces.

The normalized time-trace, V_{DEER}, as depicted in Figure S1a) is related to the distance distribution, P(r), by

$$V_{DEER} = ln\frac{V(t)}{V(0)} = \int_{r_{min}}^{r_{max}} K(r,t)P(r)dr$$

with

$$K(r,t) = -\lambda\left(1 - \int_{0}^{\pi/2} \cos(\omega_{DD}(\vartheta,r)t)\sin\vartheta\, d\vartheta\right)$$

The dipolar coupling frequency, ω_{DD}, thereby is related to the distance, r, between two paramagnetic centers by:

$$\omega_{DD} = \frac{\mu_0 g_i g_j \beta_e^2}{4\pi\hbar r^3}(3cos^2\vartheta - 1)$$

ϑ is the polar angle between the direction of the static magnetic field and the vector interconnecting the spins and all other variables have their usual meaning. Ideally, distance distributions ranging from approx. 1.5 to 8 nm can be obtained by DEER. P(r) was determined by a Tikhonov Regularization with the regularization parameter, α, of typically 100. The fit is shown in Figure S1a).

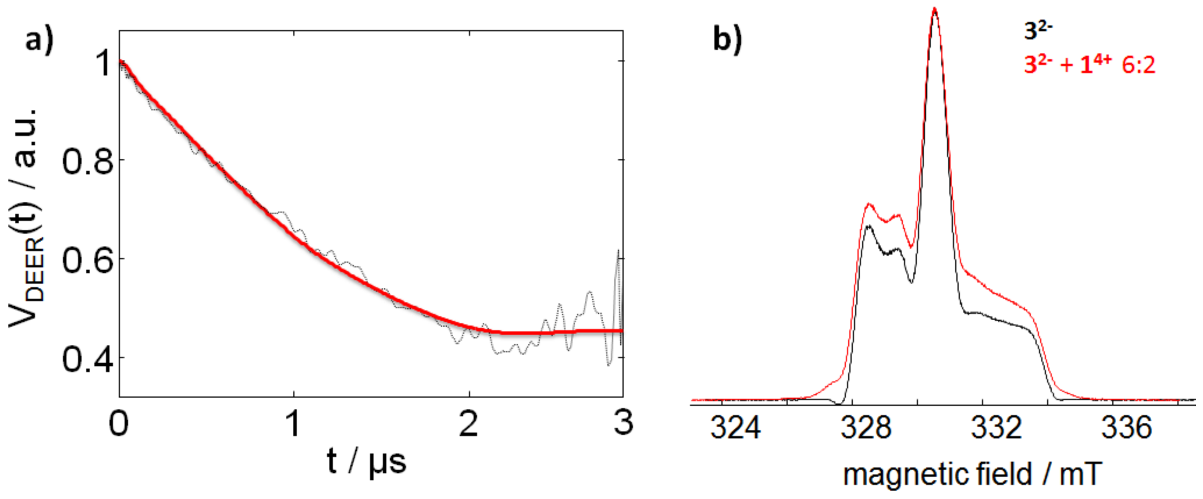

Figure S1. a) background corrected DEER signal of a solution of 6 mM $\mathbf{3}^{2-}$ and 2 mM $\mathbf{1}^{4+}$. The red line corresponds to a fit gained from Tikhonov regularization with a regularization parameter of 100. b) The field-swept EPR spectrum of $\mathbf{3}^{2-}$ (black) and $\mathbf{3}^{2-}/\mathbf{1}^{4+}$ 6:2 (red) in DMSO / 88% glycerol at 50K is depicted. The $\mathbf{3}^{2-}/\mathbf{1}^{4+}$ 6:2 spectrum appears slightly broadened, accounting for a close-contact confinement of $\mathbf{3}^{2-}$ due to the presence of $\mathbf{1}^{4+}$.

Sample Preparation

Sample preparation and compounds: All compounds were commercially available or synthesized as reported earlier.[4] Samples for EPR were prepared as follows: 6 mM $\mathbf{3}^{2-}$ were dissolved in a 1:1 mixture of DMSO and 88% glycerol. Afterwards, 2 mM of $\mathbf{1}^{4+}$ were dissolved in the $\mathbf{3}^{2-}$ solution. The final mixture was subsequently transferred to 3 mm outer diameter quartz tubes. For DEER the samples were additionally freeze-quenched in super-cooled iso-pentane. For DLS 3 mM of $\mathbf{2}^{2-}$ was dissolved in the solvent mixture and 1 mM of $\mathbf{1}^{4+}$ were added subsequently.

DLS Measurements

Our DLS setup consisted of an Ar^+ laser, operating at a wave length λ = 532 nm, as light source, an ALV goniometer to adjust the scattering angle from 50° to 110° in steps of 20°, and a fiber optic detector to measure the scattered intensity as a function of time. A commercial two lense setup (ALV) was used to reduce the natural width of the laser beam to a diameter of about 1 mm, the beam shining parallel on the sample. Intensity time correlation functions have been obtained from the homodyne detected scattered intensity using an ALV 5000 hardware crosscorrelator. All samples have been purified from dust by filtration with LCR membrane syringe filters, pore-size 0.45 µm, and put into cylindrical Suprasil light scattering cuvettes of diameter 10 mm. During the measurement, the samples were placed in a toluene bath of adjustable temperature to avoid diffraction from the glass walls of the cuvettes, and to keep the sample temperature constant at T = 20°C. From the time intensity correlation function $g_2(q,\tau)=\langle I(q,t)I(q,t+\tau)\rangle$, measured at various scattering angles, the intermediary scattering function was determined using the Siegert relation ($F_s(q,\tau)=(g_2(q,\tau)-1)^{0.5}$), with $q=4\pi n_D \sin(\theta)/\lambda$ the scattering vector and 2θ the scattering angle. This correlation function was fitted to a single-exponential decay

$F_s(q,\tau) = A \cdot \exp(-Dq^2\tau)$ to determine the diffusion coefficient $D = \frac{kT}{6\pi\eta R_H}$ given by the Stokes-Einstein-equation, and the corresponding hydrodynamic particle radius R_H.

Molecular Dynamics Simulations

We have run molecular dynamics simulations with the aim of detecting aggregating interactions of Fremy's salt and $\mathbf{1}^{4+}$. Given the available computational resources, all atom simulations covering the entire distance range accessible by DEER measurements at the nominal concentration of $\mathbf{1^{4+}}$ (2 mM) were too demanding. We have thus decided to focus on more concentrated solutions containing 18 mM $\mathbf{3^{2-}}$ and 6 mM $\mathbf{1}^{4+}$ in DMSO. The MDynaMix package was used in parallel (48 CPUs). NVT simulations were carried out on a system containing 2 $\mathbf{1}^{4+}$, 6 $\mathbf{3^{2-}}$, 8 PF_6^-, 12 K^+, and 4695 DMSO molecules confined to a cubic box of length 82.42 Å. The density of the system (1.10 g/cm3) was chosen to equal the density of DMSO at room temperature. Periodic boundary conditions were used. For electrostatic interactions, the Ewald summation approach was employed using a real-space cutoff of 15 Å and the convergence parameter α = 3 Å-1 (the standard deviation, σ, of the Gaussian charge distribution used in the Ewald approach amounts to σ = 1/(21/2 α)). The reciprocal sum was cut off for terms with the absolute value of the argument of the exponential function exceeding 9. The double time step algorithm by Tuckerman et al. was utilized to propagate the systems. The long and the short time step amounted to 2 fs and 0.2 fs, respectively. Lennard-Jones forces computed each short-time step were cut-off at 6.5 Å. The Verlet list was updated every 10 steps. The temperature was controlled using the Nosé algorithm with a reference temperature of 300 K and a relaxation time of 100 fs. After the system was equilibrated for 200 ps, production runs were performed for 8 ns. The MD trajectories were sampled at 500 fs intervals, thus 16000 configurations were collected. To characterize the structural properties of $\mathbf{3^{2-}}$ in the presence of $\mathbf{1}^{4+}$, radial distribution function of the $\mathbf{3^{2-}}$s was calculated. The method of Theodorou and Suter was used to compute the pair correlation functions for distances up to the semi diagonal of the minimum image locus.[5] A parameterization based on the AMBER force field was used: The parameters of PF_6^- were taken over from Liu et al.;[6] for DMSO parameters established by Fox and Kollman were used.[7] The parameterization of K^+ was due to Heinzinger.[8] $\mathbf{1}^{4+}$ was parameterized based on the GAFF parameters[9] with charges determined using the RESP

approach based on the AM1 optimized (in water) structure.[10] Charges assigned to equivalent positions were averaged.

Monte Carlo Simulations

We have also run Markov chain Monte Carlo simulations addressing the distribution of the charged constituents in a continuous dielectric.[11] This study was motivated by the question of whether the Coulomb interaction in combination with excluded volume effects *alone* could give rise to self-assembled structures of the kind detected here. We have vastly simplified the system with the aim of employing large periodic boxes that significantly exceed the size of the aggregates (approximately 6 nm) and that contain many replicas of the ionic constituents. In fact, we use cubic cells of dimensions up to 26 nm that contain up to 60 molecules of $\mathbf{1}^{4+}$ or 162 molecules of $\mathbf{3}^{2-}$. This is facilitated by assuming rigid molecules that interact by the Coulomb interaction in a continuous, homogenous dielectric. In addition, we have retained the repulsive part of the Lennard-Jones potentials in order to account for the hard core repulsion. To this end, the Lennard-Jones-(12,6) potentials were shifted by ε_{ij} and cut-off at their minimum, $2^{1/6}\ \sigma_{ij}$.[12] The partial charges and other parameters of the non-bonded interactions were taken over from the MD simulations detailed above. The Lorentz-Berthelot combination rule combination rule was used for Lennard-Jones parameters of mixed types. Electrostatic interaction energies were evaluated using the smooth Particle Mesh Ewald (PME) scheme suggested by Essmann et al. with $\alpha = 1.5\ nm^{-1}$.[13] Cutoffs of the real and reciprocal sum have been chosen such that energy based error bounds (assuming a randomly distributed charges) were met.[14] In practice, for an ensemble containing 2 mM $\mathbf{1}^{\mathbf{4+}}$ and 6 mM $\mathbf{3}^{\mathbf{2-}}$ within a box of side length 26 nm e.g., a real-space cutoff of 4.3 nm and 70^3 reciprocal vectors were used. The relative permittivity was assumed to amount to 46.7 (Bjerrum length: 1.2 nm, T = 298 K). Typically 100000 configurations were generated using the Metropolis algorithm. For each Monte Carlo sweep, all particles were sequentially moved at random. Each of these trails consisted of moving the center within a square of side length 2 Δr (with uniform distribution) and rotating the molecule by a random angle from the interval $[-\Delta\varphi, +\Delta\varphi]$ about a randomly orientated axis. For each trail, the energy of the newly-generated configuration was evaluated and the move accepted or rejected based on the Metropolis criterion assuming a temperature of T = 298 K. Typically, for all particles except $\mathbf{1}^{4+}$ $\Delta r = 2$ nm and $\Delta\varphi = 67°$. For $\mathbf{1}^{4+}$, $\Delta r = 0.5$ nm was used, since otherwise, due to

its comparable large size, a low acceptance ratio resulted. In this way, an overall acceptance ratio of 0.5 to 0.6 ensued depending on the actual concentrations. To speed up the calculation of pairwise interaction energies, Verlet lists were used. Only the close neighbors stored for every particle in these lists were considered. Intermolecular contributions were generally neglected, since they incur a constant shift in energy only. The neighborhood lists were updated once per sweep utilizing a cutoff distance adapted to the radii of the involved particles. Eventually, pair correlation functions were evaluated in the same way as described for the MD simulations. The simulation was implemented in Matlab (2010b, The MathWorks, Inc., Natick, Massachusetts, United States) with energy evaluations implemented in C. The bottleneck of the simulation was the evaluation of the reciprocal-space contribution to the Ewald sum. It was calculated with the Fast Fourier Transform algorithm as implemented by the FFTW library.[15]

CW-EPR Data

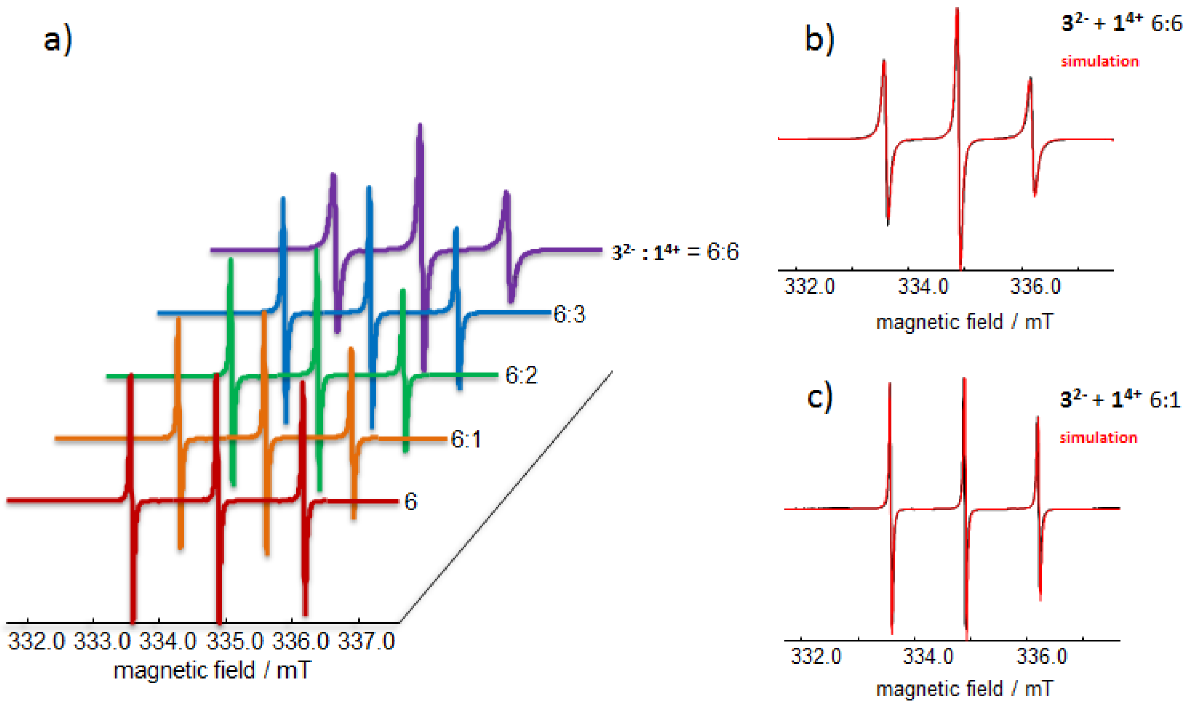

Figure S2. a) CW EPR spectra at room temperature of 6 mM $\mathbf{3}^{2-}$ and different concentrations of $\mathbf{1}^{4+}$ in 1:1 DMSO / glycerol. With increasing $\mathbf{1}^{4+}$ concentration the $\mathbf{3}^{2-}$ molecules rotate slower. b) The CW EPR spectrum of 6 mM $\mathbf{3}^{2-}$ and 6 mM $\mathbf{1}^{4+}$ is shown: A spectral simulation (red) is superimposed on the experimental spectrum (black). Applied spectral parameters for the simulation were: g_{iso} = 2.0065; A_{iso} = 36.3 MHz; D = [$1.5 \cdot 10^8$, $6.9 \cdot 10^9$] s^{-1}. c) The spectrum of 6 mM $\mathbf{3}^{2-}$ and 1 mM $\mathbf{1}^{4+}$ is shown in black. A spectral simulation is superimposed in red. Applied spectral parameters for the simulation were: g_{iso} = 2.0065 A_{iso} = 36.3 MHz D = [$6.7 \cdot 10^8$, $1.2 \cdot 10^{10}$] s^{-1}. Note that the tumbling rotation motion is not isotropic. Yet, it can be well characterized in terms of the effective rotational correlation time, which is defined as $\tau_c = 6^{-1}(D_\perp D_\parallel)^{-1/2}$, with $D_\perp$, denoting the principal components of the rotational diffusion tensor, perpendicular to the magnetic field axes and $D_\parallel$, denoting the principle components of the rotational diffusion tensor, parallel to the magnetic field axes.

The simulated rotational diffusion tensor, extractable from Figure S2b), indicates a slowed-down rotation around the molecular x- and z-axes. This implies a coordination of the two sulfonate groups to $\mathbf{1}^{4+}$. Since the molar ratio of $\mathbf{3}^{2-}$ to $\mathbf{1}^{4+}$ is 1:1, it is likely that one $\mathbf{3}^{2-}$ molecule is coordinated by only 1 $\mathbf{1}^{4+}$ pocket. With decreasing $\mathbf{1}^{4+}$ content in the solution, D becomes more isotropic, until, at a molecular ratio of 6:1, only the rotation around the

molecular z-axes is slowed down a little, compared to mere **3**$^{2-}$ in solution. This fast rotation indicates that **3**$^{2-}$ is only weakly bound to **1**$^{4+}$. Combining this knowledge with the DEER and DLS data leads to the conclusion that **3**$^{2-}$ at a ratio of 6:2 to **1**$^{4+}$ rotates quite freely, while it still populates certain points in space in the defined nano-objects, as described in the main text.

Monte-Carlo Data

K^+ and PF_6^- are, except for the region of self-avoidance close to contact (r < 4 nm), homogeneously distributed.

In the distance range from approximately 1 to 4 nm about a **3**$^{2-}$ molecule, the likelihood of observing a second **3**$^{2-}$ molecule is strongly increased. Yet, on the basis of excluded volume effects and the electrostatic interactions alone, a single peak in g(r) is observed (unlike the DEER data and the MD simulations). The peak in g(r) occurs at approximately 1.6 nm. In the mixed pair-correlation of **1**$^{4+}$ and **3**$^{2-}$, a similar peak is observed at 0.7 nm and **3**$^{2-}$ has a higher probability of being observed within a distance range of up to 5 nm about a **1**$^{4+}$ molecule than corresponded to a homogeneous distribution (not shown). Thus, **3**$^{2-}$ acts as "glue".

A surprising long-range correlation is observed for **1**$^{4+}$: Flat, yet significant, maxima occur in the range from 11 to 17 nm (see insert in Fig. 2 c)). Furthermore, both **3**$^{2-}$ and **1**$^{4+}$ are slightly depleted at a distance of approximately 18 nm. This suggest a diameter of the ionoids (for points randomly distributed on the surface of a sphere, the average mutual distance is 4/3 r, with r denoting the sphere radius) comparable to the DLS-derived hydrodynamic radii. Beyond 20 nm the sampling noise (based on 100000 snapshots) is large.

References

[1] S. Stoll, A. Schweiger, *J. Magn. Reson.* **2006**, *178*, 42-55.
[2] S. J. Schneider, J. H. Freed, *Biological Magnetic Resonance Vol. 8 - Theory and Applications*, Plenum Press, New York, **1989**.
[3] G. Jeschke, V. Chechik, P. Ionita, A. Godt, H. Zimmermann, J. Banham, C. R. Timmel, D. Hilger, H. Jung, *Appl. Magn. Reson.* **2006**, *30*, 473-498.
[4] H.-Y. Gong, B. M. Rambo, E. Karnas, V. M. Lynch, J. L. Sessler, *Nature Chemistry* **2010**, *2*, 406-409.
[5] D. N. Theodorou, U. W. Suter, *J. Chem. Phys.* **1985**, *82*, 955-967.
[6] Z. Liu, S. Huang, W. Wang, *J. Phys. Chem. B* **2004**, *108*, 12978-12989.

[7] T. Fox, P. A. Kollman, *J. Phys. Chem. B* **1998**, *102*, 8070-8079.
[8] K. Heinzinger, *Physica* **1985**, *131B*, 196-216.
[9] J. Wang, R. M. Wolf, J. W. Caldwell, P. A. Kollman, D. A. Case, *J. Comp. Chem.* **2004**, *25*, 1157-1174.
[10] C. I. Bayly, P. Cieplak, W. D. Cornell, P. A. Kollman, *J. Phys. Chem.* **1993**, *97*, 10269-10280.
[11] W. Krauth, *Statistical Mechanics: Algorithms and Computations*, Oxford University Press, Oxford, **2006**.
[12] G. S. Grest, K. Kremer, *Phys. Rev. A* **1986**, ***33****(5)*, 3628-3631.
[13] U. Essmann, L. Perera, M. L. Berkowitz, T. Darden, H. Lee, L. G. Pedersen, *J. Chem. Phys.* **1995**, *103*, 8577-8593.
[14] H. G. Petersen, *J. Chem. Phys.* **1995**, *103*, 3668-3676.
[15] M. Frigo, S. G. Johnson, *Proceedings of the IEEE* **2005**, *93*, 216-231.
